# Sulphonal

**Published:** 1890-02-22

**Authors:** 


### SULPHONAL.

In our remarks on Sulphonal in the "Retrospect" of November 23, we observed that "altogether the record of sulphonal is not very satisfactory." Additional information appears to confirm the above remark. We see Dr. Percy Smith has written to a contemporary, reporting five cases of temporary loss of power in the lower extremities occurring after the use of sulphonal. All the patients were insanes. Dr. Percy Smith remarks that, judging from the use of sulphonal by the insane, it does in some cases interfere with the cortical motor functions. Dr. Henry Sutherland, physician to the St. George's Dispensary, also contributes notes on sulphonal, and comes to the conclusion that it is a valuable sedative in chronic insanity with recurrent attacks of excitement. In continued excitement it is apparently injurious, by increasing the state of excitement on the following day. Dr. C. Knox Bond, of the City Hospital, South Liverpool, writes of the efficacy of sulphonal in the sleeplessness of typhus. It was given in twenty-three cases, and only in two did the patient become worse after sulphonal, one person became more violently delirious, and another died from collapse; but on examination of the facts, "sulphonal does not seem to have contributed to this result." The character of the sleep induced in the majority of cases was sound, natural repose.

